# Is it worth it? The costs and benefits of bringing a laptop to a university class

**DOI:** 10.1371/journal.pone.0251792

**Published:** 2021-05-24

**Authors:** Alison J. Day, Kimberly M. Fenn, Susan M. Ravizza

**Affiliations:** Department of Psychology, Michigan State University, East Lansing, Michigan, United States of America; University of Macau, MACAO

## Abstract

Students often bring laptops to university classes, however, they do not limit their laptop use to class-related activity. Off-task laptop use occurs frequently in university classrooms and this use negatively impacts learning. The present study addresses whether potential benefits of class-related laptop use might mitigate the costs of off-task laptop activity. We used tracking software to monitor both class-related and off-task laptop use by undergraduate students enrolled in an introductory psychology course, and we observed how types of laptop use related to course performance. We found a positive correlation between class-related use and exam scores that was driven by viewing lecture slides during class. We also found a negative correlation between off-task laptop use and exam scores, but class-related activities did not predict an increase in off-task use. Thus, for students who constrain their laptop use to class-related activity, the benefits outweigh the costs. While a laptop may be beneficial for some, it is unclear which students are able to constrain themselves to class-related activities and whether the benefits of class-related laptop use obtained by slide viewing could be achieved by other means. Thus, students and educators should carefully consider the costs and benefits of laptop use in the classroom.

## 1. Introduction

Over half of college students bring their laptop to class at least once a week [1,2]. The ubiquity of portable devices has sparked heated controversy and, as a result, increasing pressure is being placed on college instructors to ban laptops from their classrooms (May, 2017). On one hand, portable devices are praised as providing more diverse ways of learning, increasing student participation, and improving retention in STEM fields [3–5]. Portable devices in the classroom, however, can also provide an engaging distraction because students use laptops to perform tasks that are not related to course activities [6–9]. Indeed, these off-task activities limit students’ ability to attend and deeply process class-related information and, unsurprisingly, are associated with reduced learning in lecture-based classrooms that are typical of university education [see 10 for a review]. The potential for distraction from a laptop may be so high that students succumb to off-task activities despite their intention to use their laptop only for class-related purposes such as taking notes and viewing slides. It is currently unknown, however, whether class-related use is related to a concomitant increase in off-task laptop activity. Moreover, very few studies have used objective measures to target different types of class-related use and their relationship to learning. Evaluating this cost-benefit ratio is critical to understanding how university students are using their laptop in a typical lecture-based classroom and, consequently, whether the cost to learning from distracting laptop activities is worth the potential benefit of class-related use.

Studies investigating both class-related and off-task laptop use in lecture-based courses have largely found costs to course performance rather than benefits. For example, allowing laptop use in lecture-based, university classrooms is associated with poorer course performance compared to classrooms in which laptops were prohibited [2,10,11]. These studies, however, did not separate laptop activity into off-task and class-related use which might have obscured potential benefits from the latter. In studies examining different categories of laptop activity (e.g., social media, online shopping, and note-taking), students reported high rates of off-task use and this measure was related to poorer performance on course exams [6,7,12]. Although Fried [12] probed students about class-related laptop use (i.e., note-taking activity), the author did not report the relationship between note-taking and course performance, so it is unclear whether some aspects of laptop use were beneficial. Thus, the extensive literature on self-reported, laptop use in lecture-based courses reveals consistently negative effects on course performance, but potential benefits of laptop use remain unclear.

In fact, only two studies have targeted potential benefits of unstructured laptop activity using objective measures, and both studies reported null results [7,8]. While these studies suggest that laptop use has no benefit to learning in lecture-based classrooms, the limitations of these studies prevent strong conclusions because the effects from different types of class-related laptop use were not measured [7,8]. Different types of class-related laptop activities might be related to learning in different ways; for example, viewing lecture slides might engage the student more in the lecture whereas Internet searches on course material might distract the student. Moreover, some types of class-related laptop use, such as taking notes with a laptop, might even be detrimental to learning [13]. Perhaps unsurprisingly then, neither study found a relationship, positive or negative, between class-related use and learning. Without measuring each activity separately, it is impossible to fully understand the relationship between class-related activities and course performance. The present study remedied this problem by tracking multiple types of class-related laptop activities including slide viewing, taking notes, answering in-class questions, and viewing reference materials.

Research touting the positive aspects of computer use for classroom learning has focused primarily on highly-structured computer use. Structured laptop use (e.g., instructor-directed use to complete assignments, or to engage in a class activity) has been shown to facilitate learning in primary [14,15] and higher education [16]. For example, incorporating laptops into classroom activities resulted in better grades than a typical lecture-based format [16] and providing students and teachers with laptops increased student achievement [15]. Note, however, that it is unclear from many of these studies whether benefits in learning are the result of laptop use per se, the active-learning format of the class, or changes in teaching practice and student engagement [15,16]. In other words, the effect of laptop use is often confounded with changes in teaching methods that occur in response to laptop availability. One study found that teaching practices were different between classrooms with and without laptops [14]. However, when the researchers controlled for these differences in teaching methods, they still found a positive relationship between performance and laptop use. Thus, when used for instructional activities, computers may improve course performance, but it is unclear whether laptops are beneficial in classes when they are used in an unstructured, student-guided manner as is typical in the university classroom.

College students have more autonomy on whether to bring a laptop to class than primary school students and, thus, most of their laptop use will be self-directed. It is important to know whether unstructured, class-related laptop use such as viewing instructor-provided slides, taking notes, and searching for more information on a topic may be beneficial to learning. As class sizes in university courses continue to increase, laptops may improve learning because they allow students to view the lecture slides on their computers. For example, students may have difficulty viewing the slides because they are sitting in the back of the classroom and/or their view is obstructed. In addition, slides often include images and text, and according to dual coding theory, information that is processed through visual and verbal modes is better remembered [17,18]. By viewing slides on their laptop, students can receive the benefit of both modes of information transmission, whereas if they were unable to see the slides (due to distance or obstruction), they would need to encode the information only verbally. While a recent meta-analysis found no benefit to learning from supplementing lecture with PowerPoint slides [19], the authors point out that many of the studies showed a positive effect of slide use when students had access to the slides. Similarly, a lab-based study revealed that students who were given printed lecture slides while viewing a lecture presentation performed better on a test of the material than students who were not given the lecture slides [20]. The present study measured whether viewing lecture slides on a laptop was positively related to course performance as suggested by previous studies and investigated whether seating position is related to a potential benefit.

Another way that laptops might impact course performance is through note-taking. It seems intuitive that taking notes on a laptop would be more efficient than taking notes on paper because typing is faster than handwriting. As such, it is possible that learning would be better when students take notes on laptops because they can more quickly record their notes and this would free up time and resources to listen and engage with the material on a deeper level. However, prior work has shown that when students take notes on a laptop, they tend to write verbatim phrases directly from lecture as opposed to processing and synthesizing the information [13]. This type of note-taking has two potential consequences. First, students may spend more time writing notes verbatim than they would if they were taking handwritten notes. In an effort to write down exactly what the lecturer says, an individual may attend too much to the specific phrasing of a sentence and fail to encode a subsequent sentence. That is, an individual may devote too much cognitive effort to verbatim note taking and fail to fully attend to the lecture. Second, if students are focused on transcribing text, they may not focus on understanding or integrating information. As a consequence, students may only shallowly engage with the material. Thus, individuals who take notes on a laptop may put too much cognitive effort toward verbatim note taking at the expense of attending to the lecture and may therefore engage in shallower processing of the material. These factors may lead to poorer learning [21,22].

Despite strong theoretical predictions, research into note-taking medium has yielded equivocal results. The first study to investigate this phenomenon found that students who were assigned to take notes by hand during a lab-based study showed greater learning than those assigned to take notes on a laptop [13]. In contrast, a different lab-based study found that laptop note-takers outperformed individuals who took notes by hand [23]. Finally, a third study found that the effects of various note taking media depend on whether students reviewed their notes [24]. Thus, the current literature is inconclusive about whether students should use laptops to take notes. Importantly, however, the field is limited in that all studies are laboratory-based, and the question has not been tested in a course when there are actual consequences associated with the style of note-taking (i.e., course grades).

Benefits of laptop use, if found, may be outweighed by the costs to learning from off-task activities. Despite their best intentions, students may not be monitoring the extent of their device use and underestimate the time they spend off-task [25]. For example, a study of notification alerts reported that it took participants 8–9 minutes to get back to their primary task after email and instant messaging interruptions even though they intended to get back on task as quickly as possible [25]. Not all students will be able to implement control processes to inhibit off-task use and it is possible that having a laptop will promote increased off-task use. Indeed, several studies have shown that impulsivity and an inability to delay gratification are associated with heavy technology use [26,27]. Thus, it is critical to understand if there is a relationship between class-related and off-task laptop activities.

To better understand the relationship between in-class laptop use and course performance, we monitored all laptop activities–both Internet use and the use of applications (e.g. word processing programs)–to a greater degree than has been previously possible and using verifiable measures. The current work addresses three questions:

**RQ1. Do class-related laptop activities predict better learning?** We predicted that laptop-based note-taking would be negatively correlated with course performance, as in Mueller and Oppenheimer [13], and that slide use during class may be positively correlated with course performance. Based on previous studies, we did not expect to see high levels of any of the other class-related activities and, thus, did not expect any reliable relationships.

**RQ2. Will greater class-related laptop use be concomitant with greater off-task laptop use?** It is possible that students bring laptops to class with the intention of using them in a productive manner, but that simply having the laptop is a source of temptation. Thus, we expect both class-related and off-task laptop use to increase, and therefore, be positively correlated.

**RQ3. If benefits of class-related use are found, do they outweigh the costs associated with off-task use?** The known detrimental effects of laptop use may not outweigh potential benefits. We predict that potential benefits of laptop use will be modest, if found, and that the effect size will be smaller than that between off-task use and course performance.

The current study advances the field by acquiring detailed and objective measures of student-directed, class-related laptop use in a typical lecture style classroom. As such, this study is ideally situated to find potential relationships between class-related laptop activities and learning. It addresses the limitations of the previous work by including subtypes of class-related use and how these types of class-related use are related to factors such as standardized test performance and seating position, as well as course performance. A more thorough understanding of the potential benefits of class-related laptop use is particularly important because restricting laptop use has been found to decrease class attendance [28] which is a reliable predictor of course performance [29–31]. The results of the present study will provide some insight into whether laptops are a useful tool for students taking lecture-based classes by investigating how class-related, student-directed laptop use relates to learning and off-task activities.

## 2. Method

### 2.1 Participants

We recruited undergraduate students at Michigan State University enrolled in a large introductory psychology course held on Mondays and Wednesdays from 10:20 AM to 12:10 PM. We specifically recruited students who planned on taking a laptop to class regularly. Of the approximately 500 students enrolled in the course, 105 students consented to participate, and of those students, two later chose not to participate. The final sample consisted of 103 (73 women, 28 men, 2 unidentified) participants aged 18 to 25 (87.3% were 18 to 20, 12.7% were 21 to 25).

Given that our sample consisted of a greater percentage of women than would be expected of the typical university composition [32], we performed a binary logistic regression to assess whether laptop activity predicted gender. Using both class-related and off-task use as predictors, laptop use was not predictive of gender, χ^2^ = 1.50 (2), p = .472. A second binary logistic regression was also performed to estimate the relationship between gender and 17 subcategories of laptop use (see S1 Table). Jointly, these variables predicted gender, χ^2^ = 42.12 (17), p < .001, with significant coefficients reflecting differences in the duration of reading the news, watching videos, and using a laptop to do work for other classes. Thus, while there were some differences in how women and men were using their laptops for off-task activities, overall durations were comparable.

The distribution of class rank for our sample was similar to the composition of the entire class. In our sample, 39% were Freshmen (first-year students), 36% were Sophomore (second-year), 17% were Junior (third-year) and 8% were Senior (fourth-year). In the entire class, 47% were Freshman, 32% were Sophomore, 15% were Junior, and 7% were Senior. The average exam score for our sample was higher than that of the entire class (*M* = 82.29%, *SD* = 11.17 compared to *M* = 77.54.%, *SD* = 12.84). We could not formally compare our sample’s performance to that of the students who did not participate as we did not have consent to use individual test scores from non-participating students.

We recruited participants from one of the largest courses at Michigan State University and, therefore, was most likely to consist of a representative sample of university students relative to other classes offered at this institution. Students from a wide variety of majors (e.g. Kinesiology, Business, Pre-Med, Engineering) take this course as it is a requirement for many majors and serves as an elective course. Students were offered participation in the study if they planned on bringing a laptop to class, given the nature of our research questions. Students received research credit for completing this study. For this course, students were required to complete seven hours of research participation, and this study constituted four hours of participation. Thus, students were incentivized to complete this study because it was an easy option to complete their research hours (e.g. compared to going to a different building on campus and participating).

#### Sample size justification

No previous study has found a reliable relationship between class-related use and class performance so we based our power analysis on the average correlation (r = -.28) between off-task laptop use and exam score in our two previous studies [6,7]. We aimed to recruit at least 95 participants to provide a power of .8 and an alpha of .05.

### 2.2 Design

This was an observational study in which numerous computer-based activities were measured as independent variables. We use tracking software to objectively measure class-related and off-task activities. Class-related activities were note taking, slide viewing, using the internet to search for class related material (references), and answering questions posed by the instructor. Off-task activities included reading email, online shopping, using social media, instant messaging, using news websites, working on homework for other classes, watching videos, playing games, listening to music, looking at photos, accessing the RescueTime website, and using PowerPoint or word processors for purposes unrelated to the class. Finally, we included a random use category to include any activities that could not be placed in one of the aforementioned categories. We measured these variables to assess the extent to which time spent on these activities predicts course performance as measured by average exam score. This was a within-subject design insomuch as participants always engaged in multiple laptop activities. We also controlled for performance on the SAT or ACT to account for some of the variance attributable to general intelligence.

### 2.3 Procedure

Participants downloaded the computer monitoring application, RescueTime, onto their laptops, and the application monitored their activity during every lecture (unless the student turned the application off or did not use his or her computer). Over the course of the semester, we emailed two survey links to each participant. We sent the first survey before the second exam and the second survey before the fourth exam. Participants completed each survey online, in their own time. All procedures were approved by the Michigan State University Institutional Review Board.

### 2.4 Ethics statement

We obtained written informed consent from each of our participants to record their computer activity during class lectures. At the same time, participants were always able to turn off the monitoring application if they did not want to be monitored while engaging in a certain task. In other words, they could relinquish consent by turning off the software and subsequently restore consent by turning the monitoring application back on. This way, participants were always in control and retained their rights to privacy.

When we received the recorded data through the RescueTime application, participant names were included in the data file. We retained the confidentiality of participant laptop-use data by replacing the participant names with participant numbers before sending it to the coders. This way, none of the coders, who may have been our participants’ peers, had knowledge of the participants’ identities. We stored this de-identified data on our password-protected server, and it is only available to members of our lab. Finally, we destroyed the document linking participant names to their numbers. Thus, participants can no longer be linked to their data.

### 2.5 Social desirability

We informed participants that a computer application would monitor their activity on their laptops during lecture but that this information would not impact their course grade or even be seen by their instructor until after course grades were submitted. Further, the students were told that the instructor would not know which students were participating in the study, and the instructor was not in the room when students volunteered to participate. These promises were fully honored.

Students received full credit if they used their computer at some point during 85% of the 19 class periods and completed both surveys. In other words, students could “check in” to the RescueTime application at any point during class, immediately turn the application off, and still receive credit for that class period (i.e. they did not need to use it for the duration of the lecture). Therefore, it is unlikely that participating in this study made students use their computers in an atypical manner, because if a student wanted to engage in an activity without being monitored, they could simply turn the application off.

### 2.6 Materials and measures

#### 2.6.1 Computer use

We tracked participants’ computer use with the RescueTime application [33] which monitored computer applications (e.g. Microsoft Word), internet use, and utilities (e.g. calculator). More specifically, we received information about what applications or websites were being used; we did not receive more intrusive information such as conversations held on messaging applications. Participants configured RescueTime so that it would only track their activity during lecture, and we removed any activity that was tracked outside of class periods and during the ten-minute break during class. Thus, our data do not include time spent on class-related activities outside of class (e.g. viewing slides outside of the class period). RescueTime tracked the precise time that participants accessed a given application and the duration of time they spent using the application during the 100-minute class period (A sample of the data is available in the supplemental online materials). We collected data from every class period for which the participant was in attendance and logged onto the RescueTime application (maximum of 19 class periods; see supplemental materials for rates of attendance). Importantly, RescueTime only tracked time spent on an activity if participants were actively using that computer application. If the participant did not move the mouse or use the keyboard (inactive) for five minutes or more, only the time for which the participant used the mouse or keyboard was recorded. However, if the participant moved the mouse or used the keyboard *before* a full five minutes of inactive time had elapsed, all of that “inactive” time was recorded as active. In this case, it is assumed that the participant is still engaging with the material. From this information, we created our independent variables of interest as described in the Data Coding section (2.7).

#### 2.6.2 Course performance

Students completed four exams each consisting of 50 multiple choice questions. All questions had 4 or 5 possible response options and each question was worth 2 points, for a total of 100 points per exam. The exams focused primarily on material that was discussed during lecture and were not cumulative, meaning that each exam covered only material that had been discussed since the previous exam. We assessed course performance by averaging the four exams. The exam average acted as our dependent variable. To ensure reliability of our measure, we assessed the correlation between exams. Importantly, each of the exams was highly correlated with every other exam. We also computed a measure of reliability, Cronbach’s Alpha, across the four exam scores and found very high reliability, α = 0.89.

#### 2.6.3 Standardized test scores

We asked participants for permission to access their ACT and/or SAT scores and used these scores as proxy measures of general intelligence; performance on these exams is correlated with performance on general intelligence measures [34,35]. We used these scores in hierarchical regression analyses to assess the relationship between computer use and course performance over and above general intelligence. Note, however, that *these scores are not pure measures of general intelligence* [34–36], so we are likely not fully accounting for general intelligence. In the remainder of this paper, we will refer to these scores as proxy measures of general intelligence.

We obtained these scores from the university registrar. For the 14 students who had SAT scores but not ACT scores, we converted the SAT scores into ACT scores using an online score converter [37]. Scores were unavailable for an additional 14 participants. These participants were included in any analyses that did not include ACT score.

#### 2.6.4 Surveys

We administered two surveys online using Qualtrics [38]. In these surveys, we asked participants to: a) indicate where they sat in the class, and b) how they typically took notes—in a notebook or on a laptop. The reliability of the question assessing note taking behavior is presented in the results section. We also asked about seating position to get a rough estimate of how far away from the front of the classroom students were sitting. Due to the nature of the question (click on an image depicting the classroom) it is unlikely that the students were fully accurate but also unlikely that the participants were so inaccurate that our results are not meaningful. Students also estimated how often they sat in this location over the course of the semester. Finally, to determine potential effects of monitoring, we asked participants to rate how their internet use in this class compared to their use in other classes on a five-point Likert scale from “I used the internet much less in this class” to “I used the internet much more in this class”. Full surveys are available in the supplemental online material. Fourteen participants did not complete the surveys, so their data was not included in any analyses of survey data.

#### 2.6.5 Slide viewing

The instructor used Top Hat [39] to distribute lecture slides, and students primarily used this website to view the slides during class. Students could not access the lecture slides before class but could view slides presented during previous class periods. That is, slides presented during previous class periods remained available to students after each class period, but new lecture slides did not become available until the moment the professor discussed each slide. Thus, students could not look ahead in the lecture slides. They could view the slide that was currently being discussed in class and they could flip back to slides that had previously been displayed. The slides displayed on students’ computers automatically changed when the instructor moved to the next slide.

### 2.7 Data coding and analysis

RescueTime provided the application name or website URL and the document name (e.g. Application name: Microsoft Word, File name: PSY 101 notes; Website URL: wikipedia.com, Website document name: Ancient Greek Art) for each moment of laptop activity. From this information, we were able to sort each activity into one of many categories. We first coded the data based on the underlying activity that each application represented (e.g. word processors were coded as "note-taking" if the file name indicated that it was for this course; if this was not indicated, they were coded as "word processing" and were not included in the note-taking category; facebook.com was coded as social media). We further coded each data point as class-related or off-task (e.g. note-taking was categorized as class-related and social media sites were classified as off-task). If data could not clearly be categorized as class-related, we coded them as off-task. For the full coding scheme, see the supplemental materials. Our coding scheme was unlikely to be biased by our perspective given that class-related use was clearly defined as related to course material.

We trained two undergraduate research assistants to code the data. Each coder coded approximately half of the data, and we measured reliability by having each code three of the class periods that had previously been coded by the other coder. We compared the coding and found 94% reliability between coders. Coding was also checked for errors by the first author.

We computed an average duration for each category of use. To do this, we first summed the time spent on each category across the semester. We then divided by the number of lectures the participant attended to obtain the average amount of time spent on each activity per lecture (See the supplemental materials for attendance statistics). For example, if a participant attended 17 lectures and took notes 20–30 minutes each lecture, we would compute her note taking use at approximately 25 minutes per lecture. Scores were computed in this manner for each participant and then correlated with course performance.

Given our focus on class-related activity, we collapsed off-task activity across subtypes and correlated this measure with course performance (although, we provide subtype correlations in Table 2). For class-related activity, we performed four Pearson correlations between each class-related activity and course performance using Bonferroni correction to assess significance (.05/4 = p < .0125). For the relationships found to have statistically significant correlations, we additionally performed hierarchical multiple regressions to account for some of the variance in performance due to general intelligence, as measured by performance on the ACT and SAT tests. With this analysis, we can determine the extent to which each computer activity predicts performance beyond a proxy measure of general intelligence. We report Cohen’s *d* values and Pearson’s correlation coefficients (R^2^: Small = 0.04; Medium = 0.25; Large = 0.64) as measures of effect sizes [40]. All of these analyses were performed in SPSS version 25 [41].

## 3. Results

### 3.1 Does off-task activity relate to reduced learning?

**Fig 1:**
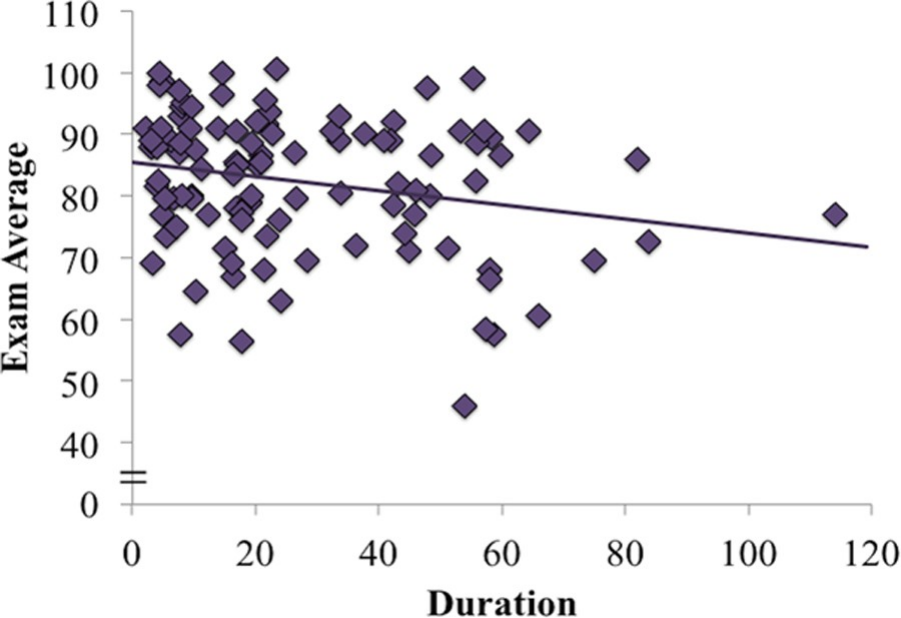
Correlation between off-task use and exam average. Durations are presented in minutes.

The means and standard deviations of the durations for each activity are presented in Table 1, and the correlations between each activity and exam score are presented in Table 2. Students spent 28% of class time using their laptops for tasks that were unrelated to the class and, as expected, off-task computer use was negatively related to course performance [6,7,12]. Average duration of off-task activity was negatively correlated with exam score, *r*(101) = -0.23, *p* = 0.02 (Fig 1). We also performed a hierarchical regression as we have done in the past [6,7]. We entered standardized test scores (as a proxy of general intelligence) in step one and off-task activity in step two and found that standardized test scores significantly predicted course performance *F*(1, 87) = 23.07, *p* < 0.001, ΔR^2^ = 0.21. Furthermore, off-task activity accounted for an additional 4.8% of the variance in exam score, *p* = 0.02, similar to what we have found in our previous studies [6,7; Table 3]. As in those studies, ACT scores were not related to the duration of off-task activity, *r*(89) = -0.12, *p* = .91. The overall model accounted for 25.8% of the variance in exam score, *F*(2, 86) = 14.93, *p* < 0.001.

**Table 1:** A. Means and standard deviations of durations (minutes) of class-related activities. B. Means and standard deviations of durations (minutes) of off-task activities.

*A*.	*M*	*SD*
**Class-related**	**34.46**	**19.43**
Note-Taking	12.51	19.61
Slides	12.30	12.15
Reference	0.36	1.98
Questions	9.28	5.47
*B*.	*M*	*SD*
**Off-task**	**27.56**	**22.25**
Email	0.96	1.10
Shopping	0.59	1.43
Social Media	0.74	1.87
Instant Messaging	1.36	2.87
News	0.47	2.06
Other Class	1.16	2.43
Video	0.32	0.94
Games	0.27	2.19
Music	0.10	0.53
PowerPoint	0.04	0.29
Word Processing	11.83	18.18
Random	7.97	7.93
Photos	0.06	0.28
RescueTime	1.68	3.47

**Table 2:** Correlations between average exam score and class-related and off-task activities.

	*r*	*P*
**Class-related**		
Note-taking	0.002	0.99
**Slides**	**0.25**	**0.01**
Reference	0.09	0.37
Questions	0.15	0.13
**Off-task**		
Email	-0.18	0.06
Shopping	-0.08	0.42
Social Media	-0.02	0.84
Instant Messaging	-0.20	0.05
News	0.12	0.25
Other Class	-0.04	0.70
Video	-0.10	0.33
Games	-0.03	0.80
Music	-0.19	0.06
PowerPoint	0.05	0.60
Word Processing	-0.18	0.06
Random	-0.01	0.94
Photos	0.04	0.69

Most of the word-processing activities classified as “off-task” were from students typing in Document1 – the default name for a Microsoft Word document. If we add these ambiguous documents to the note-taking category, the correlation with exam score changes from *r* = .002 to *r* = -.129, but is still not reliable, *r(103) = -0*.*129*, *p = 0*.*193*.

**Table 3:** Coefficients from the hierarchical regression model predicting exam score with ACT and off-task use.

	b	β	*T*
Constant	53.10	--	7.81**
ACT	1.35	0.46	4.90**
Off-task use	-0.002	-0.22	-2.35*

Note. *p < 0.05,

**p < 0.001.

Changes in off-task use also predicted changes in exam score from the first to second half of the semester. The same hierarchical regression model as described above was implemented with the addition of off-task use and exam scores from the first half of the semester in the first step of the model. Off-task use in the second half of the semester was still a significant predictor of exam scores in the second half, *F*(1, 83) = 5.23, *p* < 0.05, ΔR^2^ = 0.02.

Adding attendance to the hierarchical model, in addition to ACT, did not change the results (see supplemental results).

### 3.2 Do class-related laptop activities predict better learning?

**Fig 2:**
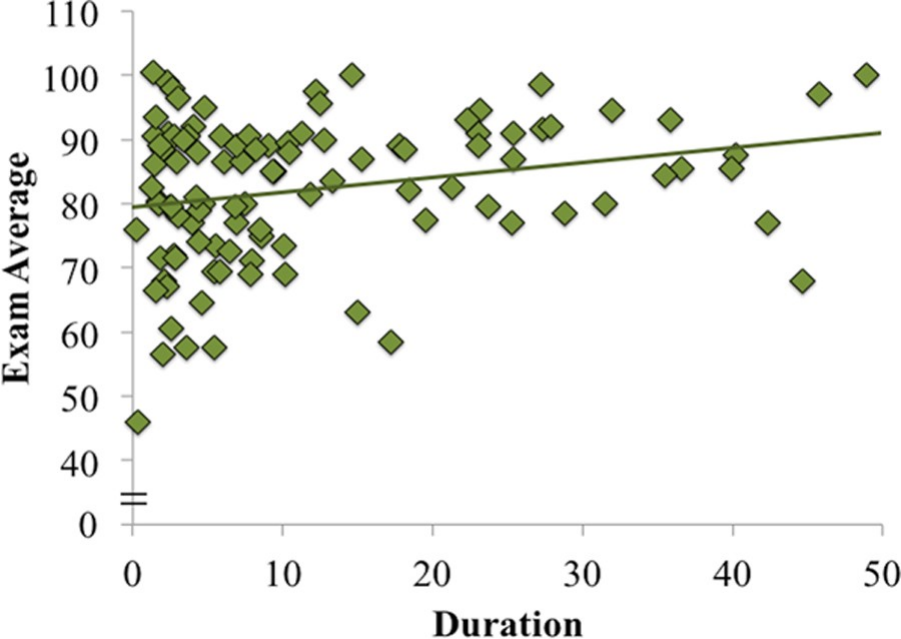
Correlation between slide use and average exam score. Durations are presented in minutes.

There were four types of activities that were categorized as class-related: note-taking, viewing lecture slides, answering questions posed by the instructor online, and using references such as Internet sites to search for class-related material. We sought to investigate the extent to which each individual activity predicted exam score and found a positive relationship between the duration of viewing slides and exam score, *r*(101) = 0.25, *p* = 0.01 (Fig 2). The other correlations were also positive, but none reached significance, *r*’s ≤ 0.15 *p*’s ≥ 0.13 (Table 2). We next used a hierarchical regression model to determine if the amount of time spent viewing slides would predict exam score over and above standardized test scores, our proxy measure of general intelligence. We entered standardized test score in step one and duration of slide viewing in step two. Standardized test scores accounted for a significant proportion of the variance in exam score, *F*(1, 87) = 23.07, *p* < 0.001. Interestingly, slide viewing contributed significantly to the model, *F*(2, 86) = 15.87, *p* < 0.001, and accounted for an additional 6.0% of the variance in exam score (Table 4). Thus, viewing lecture slides predicted course performance over and above a proxy measure of general intelligence. Indeed, ACT scores did not predict the duration of slide viewing, *r*(89) = 0.05, *p* = 0.652. Moreover, including attendance in the hierarchical model did not change the results (see the supplemental materials).

**Table 4:** Coefficients from the hierarchical regression model predicting exam score with ACT score and slide use.

	b	β	*t*
Constant	48.12	--	7.24**
ACT Score	1.32	0.45	4.83**
Slides	0.004	0.25	2.66*

Note. *p < 0.01,

**p < 0.001.

To further investigate the relationship between slide viewing and exam score, we examined the location in which participants sat in the classroom. We predicted that students who sat in the back of the classroom would be more likely to view the lecture slides on their laptops than students who sat closer to the front of the classroom because they may have had more difficulty seeing the material projected at the front of the classroom. It should be noted that the lecture hall had stadium seating so students in the back of the classroom had an unobstructed view of the screen, but they would naturally be further away than students in the front of the classroom. To examine this possibility, we asked our participants to indicate (on a map of the classroom) where they sat during a typical class period. The output of this question was in the form of continuous x-axis and y-axis variables. For seating front-to-back we used the y-axis variable where larger numbers indicated that the participants were sitting farther back in the classroom. All of the participants who completed this question indicated that they sat in this spot more than 50% of the time and 87.9% of these students indicated that they sat in this spot 90% or more of the time. Slide viewing did not correlate with participants’ front-to-back position in the classroom, *r*(90) = -0.14, *p* = 0.17, suggesting that students who sat in the back of the lecture hall did not spend more time viewing their slides during lecture.

### 3.3 Will greater class-related laptop use be concomitant with greater off-task laptop use?

**Fig 3:**
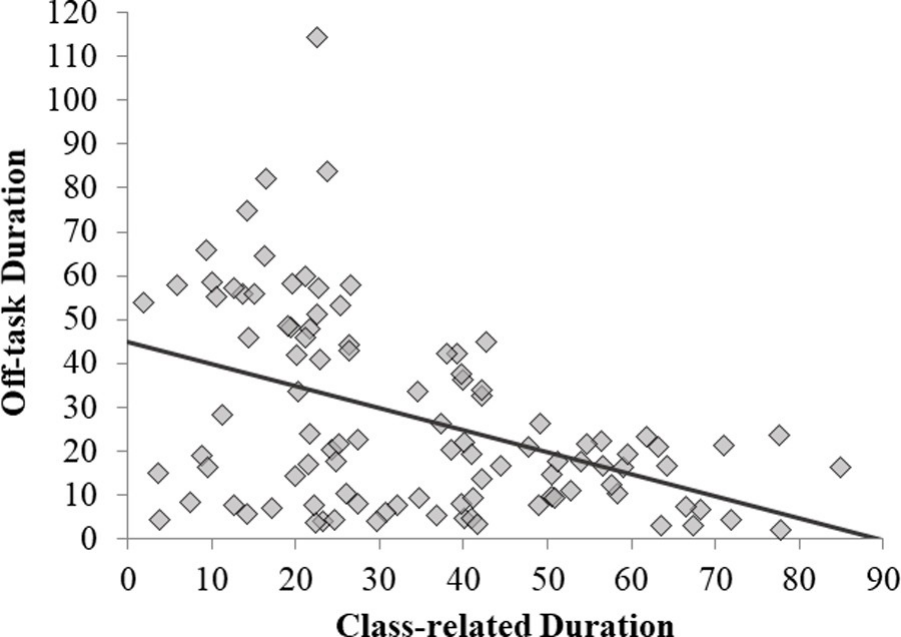
Correlation between off-task use and class-related use.

Students who use their laptop more for class-related tasks may also be more tempted to use their laptop for distracting off-task activities. This question was addressed by examining the correlation between the two types of activities (Fig 3). Note that our measures of class-related and off-task laptop activity were independent as laptop use did not take the entire class time. Thus, both positive and negative correlations were possible, and we predicted that class-related use would be positively related to off-task use. Contrary to our prediction, these variables were negatively correlated, *r*(101) = -0.44, *p* < 0.001. Thus, participants who spent more time using their laptops for class-related activities tended to spend less time doing off-task activities.

### 3.4 Do benefits of class-related use outweigh the costs associated with off-task use?

The negative relationship of off-task activity to exam score, *r* = -0.23, was similar to the effect size of the relationship between slide viewing and exam score, *r* = 0.25, *z* = 0.15, *p* = 0.88. Both effect sizes were small according to Cohen [42] and each accounted for 5–6% of the variance in exam score when accounting for general intelligence.

Thus, greater class-related use did not predict greater durations of off-task activities. Accordingly, total computer time (class-related and off-task use combined) does not contribute significantly to the model, *Δ R*^*2*^ = 0.007, *F Δ* (1, 86) = 0.718, *p* = 0.399, in the hierarchical regression of ACT and exam score. This is likely due to the opposite relationships between performance and type of computer use.

### 3.5 Social desirability

Although the students were aware that their laptop use was being monitored, our data suggest that it is unlikely that students adjusted their computer use compared to their activities in other classes. When asked how their Internet use during this class compared to their use during other classes, about 46% of students said their use was about the same as in other classes, 27.8% said they used the Internet less, and 26.7% said they used the Internet more in this class. Thus, the data was normally distributed around the median (Median = 2; Internet use was about the same). Additionally, students’ off-task use was not significantly correlated with the time they spent on the RescueTime website where they could view reports of their computer activities, *r*(101) = 0.14, *p* = 0.148.

There was some evidence, however, that off-task laptop use changed throughout the semester in response to the surveys. Surveys were given before the 2^nd^ and 4^th^ exams and we compared performance in the time periods before (i.e. before the 2^nd^ exam and between the 3^rd^ and 4^th^ exams) and after (i.e., between the 2^nd^ and 3^rd^ exams and after the 4^th^ exam) the surveys. On average, the duration of off-task activities decreased significantly by about 7 minutes in the intervals after the surveys were given, *t*(102) = 3.95, *p* < .001. The surveys may have increased social desirability effects or made students aware of the extent of their off-task activities. In either case, this pattern would have little effect on our findings. While students may have increased or decreased their off-task laptop use, the relationship to performance would remain unchanged. Indeed, negative correlations with their respective exam scores were observed both before, *r* = -0.16, and after, *r*(101) = -0.18, students completed the surveys. In other words, social desirability effects may change the duration of types of laptop activities, but not the relationship between them and exam performance.

## 4. Discussion

The present study provides the first evidence that one type of class-related laptop use predicts better learning in a lecture-based class. That is, slide viewing during class was significantly related to better course performance. Moreover, in response to our second research question, the rate of laptop use for off-task activities was not related to the amount of use for class-related activities. In fact, the more students used their laptop for class-related purposes, the less they used it for off-task activities. The relationship between exam scores and off-task or class related use were quite similar such that the cost-benefit ratio will differ depending on how much students use their laptop for each activity.

### 4.1 Duration of slide viewing predicted better exam scores

We found that accessing lecture slides during class was positively correlated with exam scores and that this relationship was independent from general intelligence defined in this research as performance on standardized tests of college readiness (ACT & SAT). In the literature targeting online access to slides, there is some disagreement about the relationship between slide use and course performance. An early study found a negative effect of slide access on course performance [43], whereas more recent studies found slide access to be beneficial to course performance [44]. Note, however, that these studies examined access to slides prior to and after lecture rather than monitoring if and how long slides were displayed on students’ laptops during class. This is the first study to demonstrate that accessing lecture slides during class has a beneficial relationship to course performance. This finding is consistent with a laboratory-based study that found that having paper handouts of slides positively impacted learning [20]. Our results show that, in addition to accessing slides, the duration of viewing slides during class is positively related to course performance.

While we predicted that slide viewing would be positively related to exam scores, we thought that it would be beneficial primarily for those sitting far from the projector screen. However, we did not find a relationship between slide viewing and seating position. Thus, it is unclear why slide viewing was related to performance. One possibility is that viewing slides on a laptop may affect how students take notes during lecture. Often, lecture slides will contain both images *and* text such that students must choose to either write down key information that the instructor says verbally or write down all of the text on the slide, or some combination of these. Recent research has found that students who have access to lecture slides tend to take better notes than those without access [20,45]. Specifically, students with access to lecture slides wrote down more critical information and topic examples [45] and wrote fewer verbatim notes (notes copied directly from the lecture slides) than students without access to slides [20]. It is possible that when students have access to slides, they actively process information and engage with the material more deeply, instead of simply transcribing text or speech. These changes in note taking likely reflect deeper information processing, which leads to greater learning [21,22,46].

Alternatively, it is possible that students who spent time viewing slides during class also spent more time viewing them outside of class. Students did not have to be in attendance to access the lecture slides, although we only measured slide viewing that occurred in the classroom. Thus, future research could adjudicate between these hypotheses by examining the content of notes taken during lecture and the amount of time spent viewing slides outside of class.

Another related and open question is whether viewing lecture slides before class might reduce the relationship between viewing slides during class and exam score. If students have access to lecture slides before class and actively encode them, viewing slides during class may not provide any added benefit for learning the material. Based on previous work that found a positive effect of hard copy lecture slides on exam performance, we do not think this likely.

### 4.2 Cost-benefit ratio depends on the student

While there was a modest benefit to exam scores related to the duration of viewing slides, we also observed a cost related to off-task laptop activities. Indeed, the time spent off-task predicted course performance even after accounting for performance on standardized tests, consistent with previous findings [6,7,12]. This result is likely due to the negative consequences of multitasking. If students engaged in off-task use while simultaneously listening to a lecture, their attention was divided amidst multiple tasks. Dividing attention leads to poorer learning [47–49]. Thus, students who spend class time doing off-task activities may not have given their full attention to the lecture and this was related to poorer learning.

In deciding an evidence-based policy about laptops in university classrooms, it is important to determine whether potential benefits will incur an equal or greater cost to learning. We predicted that students would be more tempted to use their laptop for off-task activities if using it more for class-related tasks. Instead, we found the opposite; that is, using a laptop for class-related activities was related to reduced use of distracting activities. For those using their laptop primarily for class-related use, there is a modest benefit to exam score which does not incur an equal cost to performance because of an increase in distracting laptop use. On balance, those students have a low cost-benefit ratio because they are less likely to use their laptops for off-task activities.

Conversely, students who are mostly using their laptops for off-task activities are getting little benefit associated with class-related activities and are incurring a high cost to their exam score. It cannot be determined from our study which students will be distracted by bringing their laptop to class. One possibility is that individuals with poor impulse control may have trouble resisting off-task activity if they have access to a laptop [26,50,51]. Future studies should investigate the relationship between class-related and off-task use amongst participants with high and low impulse control.

If laptop use could be restricted to class-related tasks, then there might be some benefit from bringing a laptop to class. In a higher education setting, however, this would be very difficult to enforce without invasive software or diligent monitoring. Moreover, there is no evidence that slide viewing needs to be done on a laptop for benefits to be observed. A previous study reported that having access to paper handouts of the lecture slides resulted in fewer verbatim notes than not having access to these handouts [20]. On the other hand, printing slides might be more wasteful and costly than a digital copy unless students are also taking notes on the paper copy. More research is needed to understand why slide viewing is helpful in class and whether slides need to be digital.

### 4.3 Limitations

This is a correlational study and we therefore cannot determine causality from our methods as we could with an experimental design. It is therefore possible that a third variable could underlie our primary finding. For example, an alternative explanation of the relationship between slide viewing and test scores might be that attentive students were both more likely to spend time looking at slides and to do better on tests. In other words, our findings could be a product of our sample as opposed to a product of class-related computer use.

Moreover, the characteristics of our sample raise concerns about generalizability to other student populations. Our sample slightly outperformed the rest of the class on the exams and consisted primarily of women. Due to our small sample size of men, it is unclear whether men’s computer use would correlate with course performance in the same way as it does in our entire sample. However, computer activity and course performance averages did not differ between women and men suggesting that each group engaged similarly with their laptops.

Our sample consisted of ~20% of the class who were willing to have their computer use monitored. It is possible that our sample was biased towards students who intended to use their laptops for class-related purposes. If so, off-task computer use may be underestimated compared to the general student population. Moreover, five students turned off the tracker in one class session each for an average of 36 minutes (range 2.3min– 98.5min). As it is likely that the tracker was turned off to perform off-task activities, the durations of these activities are probably underestimated for these students. Nevertheless, we observe substantial off-task activity and replicate our previous findings that such activity is inversely related to exam performance (Ravizza, Hambrick, & Fenn, 2014; Ravizza, Uitvlugt, & Fenn, 2017).

We adopted a conservative coding strategy to ensure that no off-task computer use ended up in the class-related category. For example, it is possible that students used instant messaging applications to discuss the class materials with their peers (this activity performed outside of class time has been found to improve learning; [52]). However, our data did not include information about conversations held on instant messaging applications, so we could not include any of this use in the class-related category. Although we do not think it is likely that participants used the instant messaging applications to discuss class material, if participants were engaging in this activity, it might weaken the correlation between instant messaging and learning; however, a significant negative relationship is still observed between instant messaging and exam performance, *r*(101) = -0.198, *p* = 0.045. Additionally, some social media and video use could have been class-related, but there was not enough information to code them as such. When we removed instant messaging use and ambiguous video and social media use from the off-task use category, this correlation, *r*(101) = - 0.21, *p* = 0.03, did not differ statistically from the original correlation between off-task use and course performance, *r*(101) = -0.23, *p* = 0.02, and remained significant. Thus, this use does not significantly inflate our off-task use correlation. Nonetheless, it is possible that we did not assess all of the class-related use in our data.

This study did not assess the benefits and costs of laptop use for people with disabilities or for those who have an accommodation due to poor vision or motor injury. Therefore, we cannot draw any conclusions about the utility of laptops in higher education classrooms in these populations.

## 5. Conclusion

This is the first study to show that the duration of class-related laptop use is related to better course performance. Importantly, class-related laptop use was negatively related to off-task use, suggesting that simply having a laptop in class does not increase distracting behaviors. However, the modest benefit of class-related use to exam scores was counterbalanced by the equal relationship of distracting activities to performance. If students constrain their laptop activity during class to class-related activities such as viewing lecture slides, then contrary to prior findings, laptops may be beneficial to student success in a lecture-based, university classroom. It is unclear, however, which students would benefit from bringing a laptop to class and which students would suffer. Moreover, it is unclear whether class-related tasks need to be performed on a laptop to incur the benefits. Given our results, one might consider whether the benefits of bringing a laptop to class outweigh the costs.

## Supporting information

S1 Table Two-tailed independent samples t-tests comparing exam average and each type of computer use between women and men. (DOCX)

S2 Table Attendance rates across the semester (out of 19 total classes). (DOCX)

S3 Table Data from one class period for one participant. (DOCX)

S4 Table General coding scheme. (DOCX)

S5 Table Results of hierarchical regression controlling for both ACT scores and attendance. (DOCX)

S1 Survey (DOCX)

S2 Survey (DOCX)
