# Supplementary material for: Is it worth it? The costs and benefits of bringing a laptop to a university class
Source: PLoS One. 2021 May 24;16(5):e0251792. doi: 10.1371/journal.pone.0251792 (PMC8143381; doi:10.1371/journal.pone.0251792)
Supplement: S1 Table — (DOCX) [file pone.0251792.s001.docx]

|  | Sex | *n* | *M* | *SD* |
| --- | --- | --- | --- | --- |
| Exam Average | Female | 73 | 82.110 | 11.906 |
|  | Male | 28 | 82.304 | 8.983 |
| Class-Related | Female | 73 | 2166.446 | 1208.374 |
|  | Male | 28 | 1860.682 | 1063.438 |
| Off-task | Female | 73 | 1644.352 | 1453.849 |
|  | Male | 28 | 1725.899 | 1024.030 |
| Note-taking | Female | 38 | 1551.256 | 1343.008 |
|  | Male | 12 | 1530.499 | 1084.315 |
| Reference | Female | 42 | 42.007 | 183.492 |
|  | Male | 14 | 30.725 | 36.749 |
| Slides | Female | 73 | 755.507 | 761.107 |
|  | Male | 28 | 672.182 | 671.072 |
| Questions | Female | 73 | 579.266 | 353.927 |
|  | Male | 28 | 517.210 | 255.779 |
| Email | Female | 66 | 66.165 | 74.738 |
|  | Male | 26 | 60.098 | 40.015 |
| Games | Female | 2 | 43.567 | 58.784 |
|  | Male | 3 | 473.165 | 733.031 |
| Instant Messaging | Female | 47 | 146.445 | 208.280 |
|  | Male | 18 | 82.954 | 184.069 |
| Music | Female | 14 | 10.168 | 15.873 |
|  | Male | 7 | 63.012 | 109.747 |
| News | Female | 15 | 15.201 | 15.993 |
|  | Male | 15 | 173.264 | 289.168 |
| Other Class | Female | 55 | 95.318 | 162.178 |
|  | Male | 18 | 106.019 | 179.130 |
| Photos | Female | 14 | 25.419 | 40.458 |
|  | Male | 4 | 10.249 | 8.873 |
| Random | Female | 68 | 208.361 | 272.991 |
|  | Male | 27 | 259.498 | 538.561 |
| Shopping | Female | 41 | 73.893 | 122.369 |
|  | Male | 14 | 42.468 | 47.672 |
| Social Media | Female | 32 | 88.216 | 115.058 |
|  | Male | 12 | 142.219 | 229.501 |
| Video | Female | 25 | 37.206 | 74.865 |
|  | Male | 15 | 66.373 | 95.100 |
| Internet | Female | 73 | 720.281 | 636.893 |
|  | Male | 28 | 882.414 | 782.434 |
| RescueTime | Female | 70 | 90.046 | 200.497 |
|  | Male | 26 | 154.609 | 245.345 |
