# Supplementary material for: Is it worth it? The costs and benefits of bringing a laptop to a university class
Source: PLoS One. 2021 May 24;16(5):e0251792. doi: 10.1371/journal.pone.0251792 (PMC8143381; doi:10.1371/journal.pone.0251792)
Supplement: S2 Table — (DOCX) [file pone.0251792.s002.docx]

***S2 Table.*** Attendance rates across the semester (out of 19 total classes)

| # of Classes Attended | % of Sample |
| --- | --- |
| 16-19 | 53.4% |
| 12-15 | 17.5% |
| 8-11 | 10.7% |
| 4-7 | 13.6% |
| 1-3 | 5.9% |
