# Supplementary material for: Is it worth it? The costs and benefits of bringing a laptop to a university class
Source: PLoS One. 2021 May 24;16(5):e0251792. doi: 10.1371/journal.pone.0251792 (PMC8143381; doi:10.1371/journal.pone.0251792)
Supplement: S3 Table — (DOCX) [file pone.0251792.s003.docx]

| ***S3 Table****.* Data from one class period for one participant. | | | |  |  |
| --- | --- | --- | --- | --- | --- |
| **Timestamp** | **Activity Name** | **Document Name** | **Category Name** | **Duration (sec)** | **Duration (hrs)** |
| 10/9/17 10:15 | origins.com | No Details | Business / Intelligence | 266 | 0.074 |
| 10/9/17 10:15 | msu-psychology.sona-systems.com | Studies | Uncategorized / Uncategorized | 16 | 0.004 |
| 10/9/17 10:20 | microsoft word | Document1 | Design & Composition / Writing | 251 | 0.070 |
| 10/9/17 10:20 | origins.com | No Details | Business / Intelligence | 29 | 0.008 |
| 10/9/17 10:20 | app.tophat.com | Top Hat | Reference & Learning / General | 11 | 0.003 |
| 10/9/17 10:25 | microsoft word | Document1 | Design & Composition / Writing | 185 | 0.051 |
| 10/9/17 10:25 | schedule.msu.edu | MSU Schedule of Courses : Schedule - Grid View | Reference & Learning / General | 84 | 0.023 |
| 10/9/17 10:30 | microsoft word | Document1 | Design & Composition / Writing | 267 | 0.074 |
| 10/9/17 10:30 | schedule.msu.edu | Search for Courses | Reference & Learning / General | 16 | 0.004 |
| 10/9/17 10:30 | app.tophat.com | Top Hat | Reference & Learning / General | 12 | 0.003 |
| 10/9/17 10:35 | microsoft word | Document1 | Design & Composition / Writing | 300 | 0.083 |
| 10/9/17 10:40 | microsoft word | Document1 | Design & Composition / Writing | 300 | 0.083 |
| 10/9/17 10:45 | app.tophat.com | Top Hat | Reference & Learning / General | 118 | 0.033 |
| 10/9/17 10:45 | microsoft word | Document1 | Design & Composition / Writing | 100 | 0.028 |
| 10/9/17 10:45 | mail.google.com | Shipping Confirmation - Gmail | Communication & Scheduling / Email | 43 | 0.012 |
| 10/9/17 10:45 | safari | Favorites | Utilities / Browsers | 18 | 0.005 |
| 10/9/17 10:45 | wwwapps.ups.com | Tracking: UPS | Shopping / General | 14 | 0.004 |
| 10/9/17 10:50 | microsoft word | Document1 | Design & Composition / Writing | 300 | 0.083 |
| 10/9/17 10:55 | microsoft word | Document1 | Design & Composition / Writing | 300 | 0.083 |
| 10/9/17 11:00 | microsoft word | Document1 | Design & Composition / Writing | 297 | 0.083 |
| 10/9/17 11:05 | microsoft word | Document1 | Design & Composition / Writing | 300 | 0.083 |
| 10/9/17 11:10 | microsoft word | Document1 | Design & Composition / Writing | 287 | 0.080 |
| 10/9/17 11:10 | messages | Messages (3 unread) | Communication & Scheduling / Instant Message | 13 | 0.004 |
| 10/9/17 11:15 | microsoft word | Document1 | Design & Composition / Writing | 267 | 0.074 |
| 10/9/17 11:15 | messages | Messages (2 unread) | Communication & Scheduling / Instant Message | 29 | 0.008 |
| 10/9/17 11:20 | microsoft word | Document1 | Design & Composition / Writing | 300 | 0.083 |
| 10/9/17 11:25 | microsoft word | Document1 | Design & Composition / Writing | 300 | 0.083 |
| 10/9/17 11:30 | microsoft word | Document1 | Design & Composition / Writing | 300 | 0.083 |
| 10/9/17 11:35 | microsoft word | Document1 | Design & Composition / Writing | 259 | 0.072 |
| 10/9/17 11:35 | microsoft word | No Details | Design & Composition / Writing | 23 | 0.006 |
| 10/9/17 11:35 | messages | Messages (4 unread) | Communication & Scheduling / Instant Message | 18 | 0.005 |
| 10/9/17 11:40 | microsoft word | Document1 | Design & Composition / Writing | 267 | 0.074 |
| 10/9/17 11:40 | messages | Messages (4 unread) | Communication & Scheduling / Instant Message | 33 | 0.009 |
| 10/9/17 11:45 | microsoft word | Document1 | Design & Composition / Writing | 293 | 0.081 |
| 10/9/17 11:50 | origins.com | No Details | Business / Intelligence | 162 | 0.045 |
| 10/9/17 11:50 | microsoft word | Document1 | Design & Composition / Writing | 117 | 0.032 |
| 10/9/17 11:50 | app.tophat.com | Top Hat | Reference & Learning / General | 16 | 0.004 |
| 10/9/17 11:55 | loginwindow | Login Panel | Utilities / General | 30 | 0.008 |
| 10/9/17 11:55 | app.tophat.com | Top Hat | Reference & Learning / General | 13 | 0.003 |
