# Supplementary material for: Is it worth it? The costs and benefits of bringing a laptop to a university class
Source: PLoS One. 2021 May 24;16(5):e0251792. doi: 10.1371/journal.pone.0251792 (PMC8143381; doi:10.1371/journal.pone.0251792)
Supplement: S4 Table — (DOCX) [file pone.0251792.s004.docx]

***S4 Table***. General coding scheme

| **Class Related (CR)** | | **Examples of activities** | |
| --- | --- | --- | --- |
|  | *Note taking* | docs.google.com | Evernote |
|  |  | Microsoft Word | Winword |
|  |  | Pages | Notes |
|  |  |  |  |
|  | *Slides* | Tophat | Preview |
|  |  | Powerpoint | D2L |
|  |  |  |  |
|  | *Reference* | Wikipedia | Google |
|  |  | Dictionary websites | D2L |
|  |  | Youtube etc. |  |
|  |  |  |  |
|  | *Questions* | Tophat |  |
|  | |  |  |
| **Off-Task Use** | |  |  |
|  | *Social Media* | Twitter | imgur |
|  |  | instagram | facebook |
|  |  | reddit |  |
|  |  |  |  |
|  | *Email* | mg.mail.yahoo.com | outlook.office.com |
|  |  | mail.google.com | outlook.office365.com |
|  |  | mail.msu.edu | outlook.live.com |
|  |  | microsoft outlook | login.microsoftonline.com |
|  |  | mail | login.live.com |
|  |  |  |  |
|  | *Instant Messaging* | Messages | login.yahoo.com |
|  |  | Whatsapp | Line_app |
|  |  |  |  |
|  | *News* | Espn.com | msn.com |
|  |  | Bleacherreport.com | pigeonsandplanes.com |
|  |  | Christianpost.com | squawka.com |
|  |  | Cnn.com | google.com |
|  |  | Money.cnn.com | gyazo.com |
|  |  | Football.fantasysports.yahoo.com | wunderground.com |
|  |  |  |  |
|  |  |  |  |
|  | *Other Class related* | Cliffsnotes.com | PowerPoint |
|  |  | Docs.google.com | Microsoft word |
|  |  | Google.com | Stickies |
|  |  | Paperstarter.com | Sparknotes.com |
|  |  | Preview | D2L |
|  |  | Pages |  |
|  |  |  |  |
|  | *Shopping* | Amazon.com | Etsy |
|  |  | Appstore | Revolve.com |
|  |  | Department store websites |  |
|  |  |  |  |
|  | *Games* | kongregate.com |  |
|  |  |  |  |
|  | *Videos* | Youtube | Netflix |
|  |  | hulu | amazon video |
|  |  |  |  |
|  | *Music* | Spotify | pandora |
|  |  | itunes | maskedgorilla.com |
|  | *PowerPoint* | PowerPoint use that is not class-related | |
|  | *Word Processing* | Word processor use that is not class-related | |
|  | *Photos* | Photos  Photo booth | photos.google.com  microsoft.photos |
|  | *RescueTime* | rescuetime.com |  |
|  |  |  |  |
|  | *Random* | Accounts.google.com | Northstarflags.com |
|  |  | Calendar | Google.com |
|  |  | Contacts | Sona |
|  |  | Dictionary.com | Preview |
|  |  | Maps | Prb.org |
|  |  | Msu.edu | Sleepfoundation.org |
|  |  | Stickies |  |
|  |  | *Anything that cannot be classified as class-related or one of the specific categories under off-task use. | |
|  |  |  | |
