# Supplementary material for: Is it worth it? The costs and benefits of bringing a laptop to a university class
Source: PLoS One. 2021 May 24;16(5):e0251792. doi: 10.1371/journal.pone.0251792 (PMC8143381; doi:10.1371/journal.pone.0251792)
Supplement: S5 Table — (DOCX) [file pone.0251792.s005.docx]

S8 Table. Output from the hierarchical regression models analyzing the relationships between off-task computer use or slide use and exam score. In each model, the assessed computer activity accounted for a significant portion of the variance in exam score after controlling for both ACT score and attendance. A. Off-task Use. B. Slide use.

| Step | Predictor | b | *SE* | β | *t* | *p* | R^2^ | Δ R^2^ | *F* | *df_Num_* | *df_Den_* | *p* |
| --- | --- | --- | --- | --- | --- | --- | --- | --- | --- | --- | --- | --- |
| 1 | Constant | 49.94 | 6.83 | - | 7.31 | - | - | - | - | - | - | - |
|  | ACT | 1.36 | 0.28 | 0.46 | 4.80 | <0.001 | 0.21 | - | 23.07 | 1 | 87 | <0.001 |
| 2 | Constant | 47.36 | 6.73 | - | 7.04 | <0.001 | - | - | - | - | - | - |
|  | ACT | 1.19 | 0.28 | 0.40 | 4.20 | <0.001 | - | - | - | - | - | - |
|  | Attendance | 0.48 | 0.19 | 0.23 | 2.45 | 0.016 | 0.26 | 0.051 | 15.19 | 2 | 86 | <0.001 |
| 3 | Constant | 50.42 | 6.77 | - | 7.45 | <0.001 | - | - | - | - | - | - |
|  | ACT | 1.21 | 0.28 | 0.41 | 4.33 | <0.001 | - | - | - | - | - | - |
|  | Attendance | 0.42 | 0.19 | 0.21 | 2.18 | 0.032 | - | - | - | - | - | - |
|  | Off-task use | -0.002 | 0.001 | -0.19 | -2.08 | 0.041 | 0.30 | 0.036 | 11.96 | 3 | 85 | <0.001 |

A.

| Step | Predictor | b | *SE* | β | *t* | *p* | R^2^ | Δ R^2^ | *F* | *df_Num_* | *df_Den_* | *p* |
| --- | --- | --- | --- | --- | --- | --- | --- | --- | --- | --- | --- | --- |
| 1 | Constant | 49.94 | 6.83 | - | 7.31 | - | - | - | - | - | - | - |
|  | ACT | 1.36 | 0.28 | 0.46 | 4.80 | <0.001 | 0.21 | - | 23.07 | 1 | 87 | <0.001 |
| 2 | Constant | 47.36 | 6.73 | - | 7.04 | <0.001 | - | - | - | - | - | - |
|  | ACT | 1.19 | 0.28 | 0.40 | 4.20 | <0.001 | - | - | - | - | - | - |
|  | Attendance | 0.48 | 0.19 | 0.23 | 2.45 | <0.016 | 0.26 | 0.051 | 15.19 | 2 | 86 | <0.001 |
| 3 | Constant | 46.13 | 6.58 | - | 7.01 | <0.001 | - | - | - | - | - | - |
|  | ACT | 1.19 | 0.28 | 0.40 | 4.29 | <0.001 | - | - | - | - | - | - |
|  | Attendance | 0.41 | 0.19 | 0.20 | 2.12 | 0.037 | - | - | - | - | - | - |
|  | Slide use | 0.003 | 0.001 | 0.22 | 2.35 | 0.021 | 0.31 | 0.045 | 12.51 | 3 | 85 | <0.001 |

B.
