# Supplementary material for: Is it worth it? The costs and benefits of bringing a laptop to a university class
Source: PLoS One. 2021 May 24;16(5):e0251792. doi: 10.1371/journal.pone.0251792 (PMC8143381; doi:10.1371/journal.pone.0251792)
Supplement: S1 Survey — (DOCX) [file pone.0251792.s006.docx]

*S5.* Survey 1

Start of Block: Laptop Use in the Classroom Survey

NetID Please Enter Your MSU email

________________________________________________________________

| Page Break |  |
| --- | --- |

Instructions Please answer the questions based on your activity in **Psych 101**. Your answers will not affect your grade in the class or impact the credit you receive for this study.

Q1 During a typical class, how much time on average (excluding the 10 min break) did you spend using a secondary device (i.e. a smartphone/tablet not monitored by RescueTime)…

|  | Minutes |
| --- | --- |

|  | 0 | 10 | 20 | 30 | 40 | 50 | 60 | 70 | 80 | 90 | 100 |
| --- | --- | --- | --- | --- | --- | --- | --- | --- | --- | --- | --- |

| To check social media (e.g., Facebook, Twitter, Instagram, etc.)? | 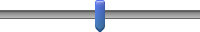 |
| --- | --- |
| To text? | 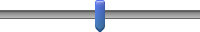 |
| To read or write email? | 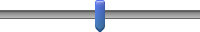 |
| To shop online? | 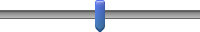 |
| To read the news or check sports scores? | 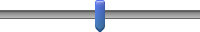 |
| To watch videos (e.g. Netflix, YouTube, etc.) | 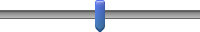 |
| To listen to music. | 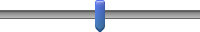 |
| To play games? | 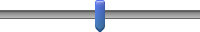 |
| To work on homework for another class? | 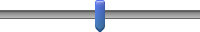 |
| For other activities? | 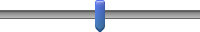 |

Q2 Did you use your smartphone/tablet for responding with TopHat?

- Yes
- No

Q3 On average, what **percent (%)** of class did you use your **laptop** for non-class related purposes?

________________________________________________________________

| Page Break |  |
| --- | --- |

Q4 How interested are you in the class?

- Very uninterested
- Somewhat uninterested
- Neither interested nor uninterested
- Somewhat interested
- Very interested

Q5 How motivated are you to do well in the class?

- Very unmotivated
- Somewhat unmotivated
- Neither motivated nor
- Somewhat motivated
- Very motivated

Q6
How many of the PSY 101 classes did you NOT attend? (starting Sept.18)

▼ 0 ... 8

Q7 In a typical class, how much time on average did you spend talking to other students during lecture?

- None at all
- A little
- A moderate amount
- A lot
- A great deal

End of Block: Laptop Use in the Classroom Survey

Start of Block: Block 1

Q8 When you used your laptop or smartphone for non-class activities, how do you think it affected your learning during class?

- It strongly helped my learning of course material
- It somewhat helped my learning of course material
- It made no difference to my learning of course material
- It somewhat disrupted my learning of course material
- It strongly disrupted my learning of course material

Q9 When you used your laptop or smartphone for class activities, how do you think it affected your learning during class?

- It strongly helped my learning of course material
- It somewhat helped my learning of course material
- It made no difference to my learning of course material
- It somewhat disrupted my learning of course material
- It strongly disrupted my learning of course material

Q10 When other students used laptops or smartphones for non-class activities, how do you
 think viewing or hearing other people’s devices affected your learning during class?

- It strongly helped my learning of course material
- It somewhat helped my learning of course material
- It made no difference to my learning of course material
- It somewhat disrupted my learning of course material
- It strongly disrupted my learning of course material
- **I never heard or saw someone else's laptop or smartphone

Q25 How often did you take notes for Psy101 on a device (or notebook) other than the one being monitored?

- Always
- Most of the time
- About half the time
- Sometimes
- Never

End of Block: Block 1

Start of Block: Block 2

Q11 How do you think your Internet usage during this class (i.e., PSY 101) compared to your peers?

- I used the Internet much less than my peers
- I used the Internet somewhat less than my peers
- About the same as my peers
- I used the internet somewhat more than my peers
- I used the Internet much more than my peers

Q12 How different was your Internet usage in this class (i.e., PSY 101) compared to your other classes?

- I used the Internet much less in this class
- I used the Internet somewhat less than my peers
- About the same as other classes
- I used the Internet somewhat more in this class
- I used the Internet much more in this class

Q13 Did you ever login to RescueTime to obtain your course credit, then disconnect immediately and continue to use your laptop unmonitored during class? NOTE: Your response to this question will NOT impact your course credit in anyway.

- Yes
- No

Q14 If you used your laptop for non-class related activities, why did you do so?

________________________________________________________________

Q15 How difficult was it to resist using your laptop for non-class activities?

- Very difficult to resist
- Somewhat difficult to resist
- Was neither difficult nor easy to resist
- Somewhat easy to resist
- Very easy to resist

Q16 How difficult was it to resist using your smartphone/tablet for non-class activities?

- Very difficult to resist
- Somewhat difficult to resist
- Was neither difficult or easy to resist
- Somewhat easy to resist
- Very easy to resist
- **I never used my smartphone or tablet in class

End of Block: Block 2
