# Supplementary material for: Is it worth it? The costs and benefits of bringing a laptop to a university class
Source: PLoS One. 2021 May 24;16(5):e0251792. doi: 10.1371/journal.pone.0251792 (PMC8143381; doi:10.1371/journal.pone.0251792)
Supplement: S2 Survey — (DOCX) [file pone.0251792.s007.docx]

*S6.* Survey 2

Start of Block: Laptop Use in the Classroom Survey

NetID Please Enter Your MSU Email

________________________________________________________________

| Page Break |  |
| --- | --- |

Instructions Please answer the questions based on your activity in **Psych 101**. Your answers will not affect your grade in the class or impact the credit you receive for this study.

Q1 During a typical class, how much time on average (excluding the 10 min break) did you spend using your monitored device…

|  | Minutes |
| --- | --- |

|  | 0 | 10 | 20 | 30 | 40 | 50 | 60 | 70 | 80 | 90 | 100 |
| --- | --- | --- | --- | --- | --- | --- | --- | --- | --- | --- | --- |

| To check social media (e.g., Facebook, Twitter, Instagram, etc.)? | 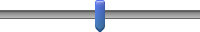 |
| --- | --- |
| To text? | 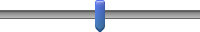 |
| To read or write email? | 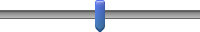 |
| To shop online? | 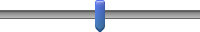 |
| To read the news or check sports scores? | 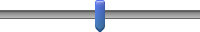 |
| To watch videos (e.g. Netflix, YouTube, etc.) | 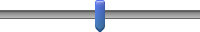 |
| To listen to music. | 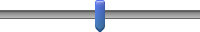 |
| To play games? | 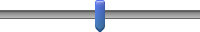 |
| To work on homework for another class? | 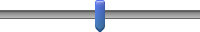 |
| For other activities? | 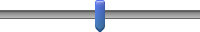 |

Q51 During a typical class, how much time on average (excluding the 10 min break) did you spend using a **secondary device** (i.e. a smartphone/tablet **not monitored** by RescueTime)…

|  | Minutes |
| --- | --- |

|  | 0 | 10 | 20 | 30 | 40 | 50 | 60 | 70 | 80 | 90 | 100 |
| --- | --- | --- | --- | --- | --- | --- | --- | --- | --- | --- | --- |

| To check social media (e.g., Facebook, Twitter, Instagram, etc.)? | 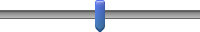 |
| --- | --- |
| To text? | 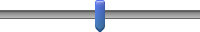 |
| To read or write email? | 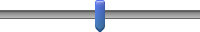 |
| To shop online? | 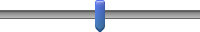 |
| To read the news or check sports scores? | 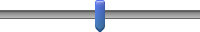 |
| To watch videos (e.g. Netflix, YouTube, etc.) | 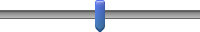 |
| To listen to music. | 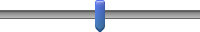 |
| To play games? | 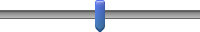 |
| To work on homework for another class? | 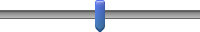 |
| For other activities? | 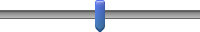 |

| Page Break |  |
| --- | --- |

Q2 Did you use your smartphone/tablet for responding with TopHat?

- Yes
- No

Q3 On average, what **percent (%)** of class did you use your **monitored** device for non-class related purposes?

________________________________________________________________

Q26 Please indicate (click on the area) where you typically sit during lectures.


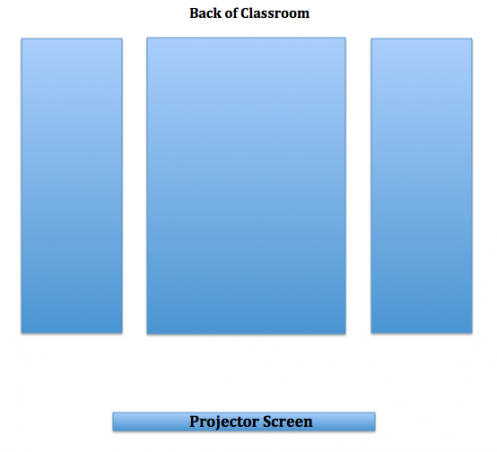


Q49 What percentage of time did you sit in this area during lecture?

________________________________________________________________

Q50 Did you sit in a different area for exams than you did for lectures?

- Always
- Sometimes
- Never

Skip To: Q48 If Did you sit in a different area for exams than you did for lectures? = Always

Skip To: Q48 If Did you sit in a different area for exams than you did for lectures? = Sometimes

Skip To: Q4 If Did you sit in a different area for exams than you did for lectures? = Never

Q48 Please indicate (click on the area) where you sat during exams. If you moved around for exams, please click in each spot (click multiple areas).


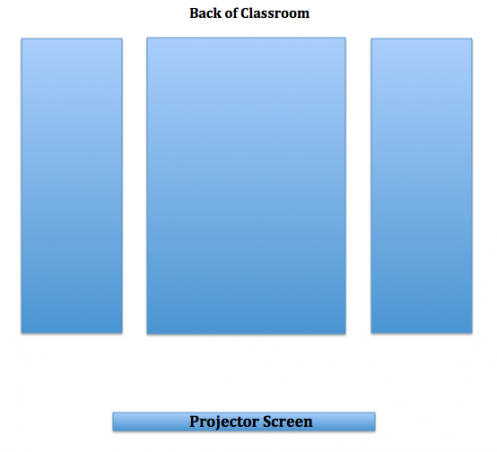


| Page Break |  |
| --- | --- |

Q4 How interested are you in the class?

- Very uninterested
- Somewhat uninterested
- Neither interested nor uninterested
- Somewhat interested
- Very interested

Q5 How motivated are you to do well in the class?

- Very unmotivated
- Somewhat unmotivated
- Neither motivated nor
- Somewhat motivated
- Very motivated

Q6
How many of the PSY 101 classes did you NOT attend? (starting Sept.18)

▼ 0 ... 8

Q7 In a typical class, how much time on average did you spend talking to other students during lecture?

- None at all
- A little
- A moderate amount
- A lot
- A great deal

End of Block: Laptop Use in the Classroom Survey

Start of Block: Block 1

Q8 When you used your laptop or smartphone for non-class activities, how do you think it affected your learning during class?

- It strongly helped my learning of course material
- It somewhat helped my learning of course material
- It made no difference to my learning of course material
- It somewhat disrupted my learning of course material
- It strongly disrupted my learning of course material

Q9 When you used your laptop or smartphone for class activities, how do you think it affected your learning during class?

- It strongly helped my learning of course material
- It somewhat helped my learning of course material
- It made no difference to my learning of course material
- It somewhat disrupted my learning of course material
- It strongly disrupted my learning of course material

Q10 When other students used laptops or smartphones for non-class activities, how do you think viewing or hearing other people’s devices affected your learning during class?

- It strongly helped my learning of course material
- It somewhat helped my learning of course material
- It made no difference to my learning of course material
- It somewhat disrupted my learning of course material
- It strongly disrupted my learning of course material
- **I never heard or saw someone else's laptop or smartphone

Q20 For this study, we monitored your internet use on your device. Most often, this was a laptop but it could have been a tablet. How often did you take notes for Psy101 on this device (laptop or tablet) versus in a notebook?

- Always on my laptop (or tablet)
- Most of the time on my laptop (or tablet)
- About half the time on my laptop (or tablet) and half the time in a notebook
- Most of the time in a notebook
- Always in a notebook

Q19 How often did you take notes for Psy101 on a device other than the one that we monitored (e.g. if we monitored your laptop how often did you take notes using a tablet)?

- Always
- Most of the time
- About half the time
- Sometimes
- Never

End of Block: Block 1

Start of Block: Block 2

Q11 How do you think your Internet usage during this class (i.e., PSY 101) compared to your peers?

- I used the Internet much less than my peers
- I used the Internet somewhat less than my peers
- About the same as my peers
- I used the internet somewhat more than my peers
- I used the Internet much more than my peers

Q12 How different was your Internet usage in this class (i.e., PSY 101) compared to your other classes?

- I used the Internet much less in this class
- I used the Internet somewhat less than my peers
- About the same as other classes
- I used the Internet somewhat more in this class
- I used the Internet much more in this class

Q13 Did you ever login to RescueTime to obtain your course credit, then disconnect immediately and continue to use your laptop unmonitored during class? NOTE: Your response to this question will NOT impact your course credit in anyway.

- Yes
- No

Q14 If you used your laptop for non-class related activities, why did you do so?

________________________________________________________________

Q15 How difficult was it to resist using your laptop for non-class activities?

- Very difficult to resist
- Somewhat difficult to resist
- Was neither difficult nor easy to resist
- Somewhat easy to resist
- Very easy to resist

Q16 How difficult was it to resist using your smartphone/tablet for non-class activities?

- Very difficult to resist
- Somewhat difficult to resist
- Was neither difficult or easy to resist
- Somewhat easy to resist
- Very easy to resist
- **I never used my smartphone or tablet in class

End of Block: Block 2

Start of Block: Block 3

Q47 In this section, we are going to list a series of phrases. Please select how much each of these phrases applies to you.

Q46 I am the life of the party

- Very inaccurate
- Moderately inaccurate
- Neither accurate nor inaccurate
- Moderately accurate
- Very accurate

Q45 I sympathize with others' feelings

- Very inaccurate
- Moderately inaccurate
- Neither accurate nor inaccurate
- Moderately accurate
- Very accurate

Q44 I get chores done right away

- Very inaccurate
- Moderately inaccurate
- Neither accurate nor inaccurate
- Moderately accurate
- Very accurate

Q43 I have frequent mood swings

- Very inaccurate
- Moderately inaccurate
- Neither accurate nor inaccurate
- Moderately accurate
- Very accurate

Q42 I have a vivid imagination

- Very inaccurate
- Moderately inaccurate
- Neither accurate nor inaccurate
- Moderately accurate
- Very accurate

Q41 I don't talk a lot

- Very inaccurate
- Moderately inaccurate
- Neither accurate nor inaccurate
- Moderately accurate
- Very accurate

Q40 I am not interested in other people's problems

- Very inaccurate
- Moderately inaccurate
- Neither accurate nor inaccurate
- Moderately accurate
- Very accurate

Q39 I often forget to put things back in their proper places

- Very inaccurate
- Moderately inaccurate
- Neither accurate nor inaccurate
- Moderately accurate
- Very accurate

Q38 I am relaxed most of the time

- Very inaccurate
- Moderately inaccurate
- Neither accurate nor inaccurate
- Moderately accurate
- Very accurate

Q37 I am not interested in abstract ideas

- Very inaccurate
- Moderately inaccurate
- Neither accurate nor inaccurate
- Moderately accurate
- Very accurate

Q36 I talk to a lot of people at parties

- Very inaccurate
- Moderately inaccurate
- Neither accurate nor inaccurate
- Moderately accurate
- Very accurate

Q35 I feel others' emotions

- Very inaccurate
- Moderately inaccurate
- Neither accurate nor inaccurate
- Moderately accurate
- Very accurate

Q34 I like order

- Very inaccurate
- Moderately inaccurate
- Neither accurate nor inaccurate
- Moderately accurate
- Very accurate

Q33 I get upset easily

- Very inaccurate
- Moderately inaccurate
- Neither accurate nor inaccurate
- Moderately accurate
- Very accurate

Q32 I have difficulty understanding abstract ideas

- Very inaccurate
- Moderately inaccurate
- Neither accurate nor inaccurate
- Moderately accurate
- Very accurate

Q27 I keep in the background

- Very inaccurate
- Moderately inaccurate
- Neither accurate nor inaccurate
- Moderately accurate
- Very accurate

Q28 I am not really interested in others

- Very inaccurate
- Moderately inaccurate
- Neither accurate nor inaccurate
- Moderately accurate
- Very accurate

Q29 I make a mess of things

- Very inaccurate
- Moderately inaccurate
- Neither accurate nor inaccurate
- Moderately accurate
- Very accurate

Q30 I seldom feel blue

- Very inaccurate
- Moderately inaccurate
- Neither accurate nor inaccurate
- Moderately accurate
- Very accurate

Q31 I do not have a good imagination

- Very inaccurate
- Moderately inaccurate
- Neither accurate nor inaccurate
- Moderately accurate
- Very accurate

End of Block: Block 3
